# Hepatitis E Virus Exposure Across Multiple Host Species in a Shared Ecosystem in Argentina

**DOI:** 10.3390/vetsci13020179

**Published:** 2026-02-11

**Authors:** Agostina Tammone Santos, Mariana A. Rivero, Walter E. Condorí, Tamara B. Soto, María C. Moran, Andrea E. Caselli, Adela Tisnés, Marcela M. Uhart, Silvina E. Gutiérrez, Silvia M. Estein

**Affiliations:** 1 Centro de Investigación Veterinaria de Tandil ((CIVETAN), UNCPBA-CICPBA-CONICET), Campus Universitario, Paraje Arroyo Seco s/n, Tandil 7000, Argentina; agostinatammone@vet.unicen.edu.ar (A.T.S.); mrivero@vet.unicen.edu.ar (M.A.R.); walter.condori@vet.unicen.edu.ar (W.E.C.); tbsoto@vet.unicen.edu.ar (T.B.S.); mcmoran@vet.unicen.edu.ar (M.C.M.); segutier@vet.unicen.edu.ar (S.E.G.); 2 Programa de Conservación Comunitaria del Territorio, Departamento de Ciencias Biológicas, Facultad de Ciencias Veterinarias, Universidad Nacional del Centro de la Provincia de Buenos Aires, Campus Universitario, Paraje Arroyo Seco s/n, Tandil 7000, Argentina; andreaca@vet.unicen.edu.ar; 3 Facultad de Ciencias Veterinarias, Universidad Nacional del Centro de la Provincia de Buenos Aires, Campus Universitario, Paraje Arroyo Seco s/n, Tandil 7000, Argentina; 4 Área de Epidemiología, Facultad de Ciencias Veterinarias, Universidad Nacional del Centro de la Provincia de Buenos Aires, Campus Universitario, Paraje Arroyo Seco s/n, Tandil 7000, Argentina; 5 Facultad de Ciencias Humanas, Universidad Nacional del Centro de la Provincia de Buenos Aires-CIG-IGEHCS-CONICET, Campus Universitario, Paraje Arroyo Seco s/n, Tandil 7000, Argentina; atisnes@fch.unicen.edu.ar; 6 Karen C. Drayer Wildlife Health Center, School of Veterinary Medicine, University of California Davis, 1089 Veterinary Medicine Dr, VM3B Ground Floor, Davis, CA 95616, USA; muhart@ucdavis.edu; 7 Laboratorio de Inmunología, Facultad de Ciencias Veterinarias, Universidad Nacional del Centro de la Provincia de Buenos Aires, Campus Universitario, Paraje Arroyo Seco s/n, Tandil 7000, Argentina

**Keywords:** invasive alien species, hepatitis E virus, One Health, zoonotic infection, serology, wildlife health

## Abstract

The hepatitis E virus (HEV) is an emerging multi-host pathogen, with suids being the main reservoir. Humans are primarily infected through the consumption of contaminated water or food. In Argentina, HEV circulation has been confirmed in humans, domestic pigs, wild boar (*Sus scrofa*), and surface water. In El Palmar National Park, invasive wild boar and axis deer (*Axis axis*) are controlled, and their meat is released for public consumption, with trimmings and offal frequently fed to dogs. Between 2017 and 2019, we conducted a multi-species serological survey in this protected area to assess HEV exposure in invasive mammals and in dog and human consumers of game meat. We also evaluated associations between seropositivity and environmental variables, as well as behavioral risk factors among game-meat consumers. Total anti-HEV antibodies were detected in 29/75 (38.67%) wild boar, 1/134 (0.75%) deer, 1/18 (5.6%) dogs, and 6/59 (10.17%) humans. A spatial cluster of seropositive wild boar was identified in a low-lying, flood-prone area near the confluence of the El Palmar stream and the Uruguay river, suggesting increased risk of environmental transmission. This is the first report of HEV exposure in wild boar from this park and in axis deer and dogs in Argentina. Participation in culling and game meat handling and consumption may contribute to HEV exposure pathways among humans. These findings improve understanding of HEV epidemiology at the wildlife–domestic animal–human interface and highlight the influence of environmental factors and human behavior on zoonotic virus circulation.

## Simple Summary

Hepatitis E virus (HEV) can infect both animals and people and is commonly transmitted through contaminated water or food. Wildlife, particularly wild boar, can help maintain the virus in the environment and create opportunities for infection across species. In a protected area of Argentina where invasive wild boar and deer are managed and their meat is consumed by people and dogs, we investigated exposure to HEV in wildlife, domestic animals, and humans who share the same ecosystem. Antibodies in blood samples showed that exposure to the virus was common in wild boar and less so in humans, dogs, and deer. In wild boar, higher exposure was detected in low-lying areas near water bodies that are prone to flooding, suggesting these environmental conditions can favor virus persistence and transmission. This study provides novel antibody-based evidence of HEV exposure in axis deer and dogs in Argentina and the first report of exposure in wild boar from this protected area. Thus, hunting practices and game meat consumption habits may increase risk of exposure in humans. These findings highlight the importance of integrating wildlife surveillance, environmental factors, and human activities into efforts to reduce the risk of diseases that can be shared between animals and people.

## 1. Introduction

*Paslahepevirus balayani*, formerly known as hepatitis E virus (HEV), is an emerging multi-host zoonotic pathogen with widespread distribution and significant public health relevance [1]. In 2021, the World Health Organization (WHO) estimated 20 million cases of acute hepatitis E globally, resulting in around 3450 deaths [2].

HEV is a single-stranded, positive-sense, non-enveloped ribonucleic acid (RNA) virus belonging to the *Hepeviridae* family, currently classified within the species *Orthohepevirus A*. [3]. HEV is an enteric virus that can be excreted in the feces of humans and reservoir animals [4]. Eight genotypes of *P. balayani* have been described, of which five genotypes (HEV-1 to 4 and HEV-7) are known to infect humans, while six have been reported to infect animals (HEV-3 to HEV-8) [5]. HEV-1 and HEV-2 have been found only in humans and are often associated with large waterborne outbreaks in low-income regions with poor sanitation and inadequate treatment of sewage [4]. In most cases, these infections are typically self-limiting and do not progress to chronic disease; however, they can cause severe acute hepatitis in pregnant women, with a 20–30% risk of maternal and fetal mortality [6]. In contrast, HEV-3 and HEV-4 are common in high-income countries and generally cause sporadic asymptomatic infection in humans. In immunocompetent individuals the fatality rate is low (around 0.5–4%) but in immunocompromised people they can lead to chronic hepatitis, hepatic failure, or extrahepatic manifestations, particularly in solid organ transplant recipients [7,8,9]. Foodborne transmission through the consumption of undercooked meat, mainly from domestic pigs (*Sus scrofa domesticus*), wild boar (*Sus scrofa*), and, to a lesser extent, cervids, and direct contact with these reservoir species represent the main source of human infection with HEV-3 and HEV-4 genotypes [10]. In addition, the ingestion of contaminated drinking water is considered another possible route of HEV-3 and HEV-4 transmission [11,12,13].

Anti-HEV antibodies have been detected in many wild animal species in different countries, including Norway rats (*Rattus norvegicus*) [14], moose (*Alces alces*), reindeer (*Rangifer tarandus*), red deer (*Cervus elaphus*) and muskoxen (*Ovibos moschatus*) [15], as well as in domestic animals such as companion animals (dogs, cats), ruminants (cows, sheep, and goats) [16], birds, rabbits and horses, suggesting that these animal species are exposed to HEV [12,17]. However, the role and relevance of these unusual host species in HEV transmission is still unclear. Nevertheless, a growing number of studies have shown that HEV-3 infections of animals in close contact with humans, including companion and farm animals, pose a risk for cross-species transmission [18,19].

In recent years, understanding the epidemiology of HEV has gained increasing attention in Latin America. Across the region, HEV is believed to be predominantly transmitted through zoonotic routes, with domestic pigs serving as a primary reservoir [20]. In Argentina, HEV-3 has been reported in pig herds, wild boar, environmental water sources, and humans [21,22,23,24,25,26]. In Uruguay, HEV-3 has also been identified in captive white-collared peccaries (*Pecari tajacu*) and axis deer (*Axis axis*) [27,28]. In Argentina and Uruguay, phylogenetic analysis has shown a high degree of homology between animal and human strains [24,25,26,29,30], suggesting zoonotic transmission.

Wild boar and axis deer are invasive alien species that have established and expanded across several regions of Argentina, causing significant ecological and health impacts, particularly within protected areas. Consequently, some national parks, such as El Palmar National Park (EPNP) in Entre Ríos, Argentina, have implemented long-term population control programs to mitigate their effects on native ecosystems [31,32]. Following negative testing for *Trichinella* spp., meat from culled animals is distributed for consumption among authorized stakeholders, including hunters, park staff, and residents from nearby communities. Additionally, dogs are frequently fed meat trimmings and offal derived from the hunted animals [33].

Given the lack of information on the presence and epidemiology of HEV in EPNP, and the potential exposure risks associated with hunting and handling of animal viscera, as well as the possibility of foodborne transmission to consumers, we conducted a serological survey for HEV in wild boar and axis deer culled at EPNP, as well as in dogs and human consumers of this game meat. In addition, demographic and spatial variables associated with seropositivity were also studied. This study provides applied epidemiological evidence to support HEV surveillance and prevention across multiple host species within a shared ecosystem.

## 2. Materials and Methods

### 2.1. Study Area

**Figure 1:**
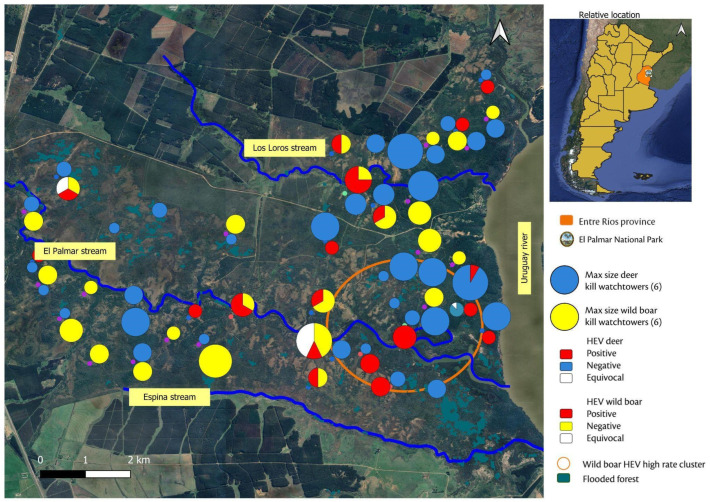
Map of Argentina showing the location of Entre Ríos province (**upper-right panel**) and the location of El Palmar National Park in this province. The left panel shows the sites where wild boar (*Sus scrofa*) and axis deer (*Axis axis*) were sampled within the park between 2017 and 2019. The location of the watchtowers is represented by circles of different sizes, according to the number of wild boar and deer killed in this area.

The EPNP (31°51′54″ S, 58°15′34″ W) covers 8500 ha [34], in the southeast of Entre Ríos, Argentina, along the margins of the Uruguay river, which forms the international boundary between Argentina and Uruguay (Figure 1). The park features predominantly flat terrain intersected by a network of streams—including El Palmar, Los Loros and Espina—that drain into the Uruguay river, which has an average annual discharge of about 4000 m^3^/s. Particularly, the El Palmar stream divides the park into two zones: the northern zone for public use includes recreational areas and trails; and the southern zone, which is restricted to authorized personnel only. The park biome consists of subtropical savanna dominated by *Butia yatay* palm groves, interspersed with grasslands, shrublands, and riparian forests, as well as patches of wetlands, lagoons, and seasonally variable marshes. The climate is humid subtropical, with hot summers (often exceeding 30 °C) and mild winters (average temperatures 8–15 °C). Annual rainfall ranges from 1200 to 1400 mm, concentrated between November and March [35].

Since 2006, a systematic control program targeting invasive ungulates—wild boar and axis deer—has been implemented by park rangers and trained local hunters [31]. Long-term monitoring data show that wild boar abundance declined markedly following program initiation, whereas axis deer abundance increased and later stabilized under sustained control effort [32,36]. Spatial analyses further indicate that wild boar captures were on average 43% higher in the restricted-use conservation zone, while axis deer captures were 60% higher in the public-use area [36]. Hunted animals are slaughtered on site, tested for *Trichinella* spp., and then distributed for consumption by hunters, park personnel, and local residents. Meat trimmings and offal are generally used to feed dogs [33].

### 2.2. Sample Collection and Analysis

This study was conducted using the same set of serum samples analyzed in previous works [33,37], collected during the same sampling period under identical field and laboratory conditions. Differences in the number of wild boar, axis deer, dog and human samples included in this study are due to insufficient sera in some cases.

#### 2.2.1. Wild Boar and Axis Deer Sampling

Between March–June and October–December 2017, and February–May 2018 and August–November 2019, blood samples from wild boar and axis deer were collected [33,37]. Wildlife samples were obtained opportunistically during routine population control and management activities conducted by park authorities. Trained hunters collected blood by jugular venipuncture in the field within 5 min after culling. Each specimen was labeled with the corresponding watchtower identification number for location, and animals were classified by species, sex, and body length [31,32]. Samples were kept at room temperature and centrifuged within 4 h, and sera were stored at −20 °C.

#### 2.2.2. Dog Sampling and Questionnaires

Dogs that were regularly fed game meat or offal from culled wild boar and axis deer from EPNP or nearby private lands were enrolled between 2017 and 2019 [33]. All procedures were approved by the Animal Welfare Committee (Acta 087/02) of the Facultad de Ciencias Veterinarias, Universidad Nacional del Centro de la Provincia de Buenos Aires (Tandil, Argentina). Written informed consent was obtained from all dog owners prior to sampling.

Blood samples (3 mL) were obtained by a veterinarian by cephalic venipuncture, maintained at room temperature, and centrifuged within 2 h of collection. Serum aliquots were stored at −20 °C until analysis. Demographic and feeding data were obtained through a structured questionnaire. Each dog was classified by sex, age, and by the frequency and type of game consumption [33].

#### 2.2.3. Human Sampling and Questionnaires

Between 26–30 August and 11–15 November 2019, individuals who consumed meat from wild boar or axis deer hunted in EPNP were enrolled [33]. The study was approved by the Comité Central de Bioética en la Práctica y en la Investigación Biomédica (Paraná, Entre Ríos, Argentina), and written informed consent was obtained from all participants prior to inclusion.

Blood samples (5 mL) were collected by cephalic venipuncture by medical personnel from Hospital Público San Benjamín (Colón, Entre Ríos), and sera were stored at −20 °C. Before sampling, participants completed a structured questionnaire that included sociodemographic and occupational information, as well as details on game meat handling, preparation, and consumption practices. Additional questions addressed prior diagnoses of acute or chronic hepatitis and other zoonotic diseases, including brucellosis, tuberculosis, leptospirosis, and trichinellosis.

#### 2.2.4. Detection of HEV Antibodies

Serum samples from invasive alien species, domestic dogs and humans were tested for the presence of total HEV antibodies (combined IgG, IgM and IgA) using the commercial kit Enzyme-Linked Immunosorbent Assay (ELISA) HEV Ab version Ultra; DIA.PRO multi species (Milan, Italy) according to the manufacturer’s instructions. All assay steps were performed manually. This is an antigen double sandwich ELISA that uses synthetic peptide encoding for conservative and immunodominant epitopes (open reading frame -ORF- ORF2 and ORF3) derived from HEV-1 and HEV-3 for coating and Horseradish Peroxidase (HRP)-labeled peptides for detection. According to the manufacturer, the reported sensitivity of this ELISA kit is 100% and the specificity is over 99.5%; these parameters were considered in previous studies conducted in wild boar and axis deer [27,38]. For each tested sample, the OD (Optical Density)/cut-off ratio was calculated. Samples with a ratio > 1.1 were considered positive, samples with a ratio < 0.9 were considered negative, and samples with a ratio 0.9–1.1 were considered equivocal. Samples with equivocal ELISA results were excluded from statistical analyses.

#### 2.2.5. Geographical Location of Hunting Posts

To establish the distribution of wild boar and axis deer, the watchtowers where the animals were culled were identified, georeferenced and assigned to either the northern or southern areas of the park. The spatial distribution of these watchtowers and the serological results for HEV were mapped using QGis 3.28.3. For each tower, the distances to different watercourses were calculated, and the altitude in meters above sea level (m.a.s.l.) was assigned based on a Digital Elevation Model (DEM). A layer of floodplain forests, available from MapBiomas [39], was included.

#### 2.2.6. Statistical Analysis

Seropositivity rates and 95% confidence intervals (CIs) were calculated for each species. Categorical and continuous variables were summarized using descriptive statistics. Associations between seropositivity and the categorical variables under investigation were evaluated using contingency table analyses with Pearson Χ2 Chi square test or Fisher’s exact test, when applicable. Odds ratio (OR) and 95% CIs were estimated for variables with a statistically significant *p* value (*p* < 0.05). For continuous variables, two-sided Wilcoxon’s rank-sum tests were used. All statistical analyses were performed using InfoStat software (v2018) [40].

#### 2.2.7. Spatial Analysis

Potential spatial clusters of high and low rates of seropositivity were investigated in the study area applying a Bernoulli model using SaTScan software, v10.0.2. [41], as an exploratory spatial approach.

## 3. Results

### 3.1. Serological Analysis and Risk Factors

#### 3.1.1. Seropositivity in Wild Boar and Axis Deer

A total of 210 samples were analyzed using a commercial HEV ELISA: 75 were taken from wild boar and 134 from axis deer. A summary of the categorical variables, the location of watchtowers where wild boars were killed, and the seropositivity to HEV are presented in Table 1. HEV-specific antibodies were detected in 29 out of 75 (38.67%; CI 95% 26.98–50.35) wild boar. Of these, 26 seropositive animals were detected in 2017 (26/60; 43.3%), while 3 seropositive animals were identified in 2018 (3/15; 20.0%). Four samples from two females and two males showed an equivocal result, all corresponding to samples collected in 2017. A greater proportion of females tested positive (15/30, 50%) than males (14/45, 31.11%), though this difference was not statistically significant (*p* = 0.0980). Additionally, no association with age category was observed (*p* = 0.9623). No significant associations were found between the HEV seroprevalence for wild boar killed in the northern and southern areas of the park (*p* = 0.2505).

For axis deer, 56 females (41.48%) and 79 males (58.52%) were sampled, including 27 juveniles (20%), 82 young adults (60.74%) and 26 old adults (19.25%). Unlike wild boar, only one adult male axis deer (1/134; 0.75%; CI 95% 0.02–4.08) killed in the northern area was seropositive to HEV. This seropositive deer was sampled in 2017 (1/102; 0.98%), whereas no seropositive animals were detected among deer sampled in 2018 (0/32). A sample from one male from 2017 showed an equivocal result.

#### 3.1.2. Spatial Analysis of Wild Boar Samples

The spatial analysis identified a cluster of high seropositivity located at the confluence where El Palmar stream discharges into the Uruguay river, a low-elevation area prone to flooding. Within this cluster, 10 of 12 individuals (83.3%) were seropositive for HEV (*p* = 0.051), suggesting a possible association between geographic factors and infection risk. Complementary statistical analyses showed that seropositive individuals tended to be located at lower mean altitudes and at shorter mean distances to the Uruguay river than seronegative ones; however, these differences were not statistically significant and are presented as descriptive patterns consistent with the spatial cluster detected (Table 2).

Spatial distribution of the watchtowers, the number of culled animals and HEV seropositivity rates at each location, the distribution of flooded forest, and the identified high-risk spatial cluster are shown in Figure 1.

#### 3.1.3. Seropositivity in Dogs

Blood samples were obtained from 18 dogs: 7 females and 11 males, including 2 pups, 1 young, and 15 adults. Owners indicated that their dogs’ diet included meat and offal from hunted wild boar and deer [33]. A total of 45 out of 59 (76%) participants in the EPNP culling program reported feeding game offal to their own dogs, to neighbors’ dogs, or to strays at high (10 to 14 per week) or medium (5 to 9 per week) frequency. Most owners (35/45, 77.8%) stated that game remains were uncooked. In all cases, game meat was supplemented with other food types (e.g., rice) at the discretion of each owner. The frequency of feeding game meat to dogs in personnel of EPNP is summarized in Table 3. Out of 18 sera tested, only one adult male was positive for HEV antibodies (1/18, 5.6%; CI 95% 0.14–27.29).

#### 3.1.4. Seropositivity in Humans

Fifty-nine participants responded to the questionnaire and provided blood samples, including 47 males (79.66%) and 12 females (20.33%) aged between 27 and 64 years. Enrolled participants included 31 hunters (52.54%), 14 park rangers (23.72%), and 14 individuals with “other” duties (23.72%) [33]. The latter group comprised firefighters (*n* = 10; 66.67%), support staff (*n* = 3; 20%), and researchers (*n* = 2; 13.33%). Housewives were classified according to their husbands’ duty categories due to shared game meat consumption habits. Questionnaire responses and participant demographics are summarized in Table 3.

None of the participants reported being previously diagnosed with acute or chronic hepatitis, nor with other zoonoses such as brucellosis, leptospirosis, tuberculosis or trichinellosis. Out of 59 individuals tested, antibodies against HEV were detected in six individuals (6/59, 10.17%; CI 95% 1.61–18.73), comprising three hunters (3/59; 5.08%), one park ranger (1/59; 1.69%), and two “other” duty category participants (2/59; 3.38%). In addition, one sample from a male park ranger showed an equivocal result. However, statistically significant differences were not found among groups (*p* = 0.8403). A greater proportion of women tested positive (3/12, 25%) than men (3/47, 6.38%), though this difference was not statistically significant (*p* = 0.0755). Additionally, no association with age was observed (Wilcoxon, *p* = 0.8380).

No significant association was found between the frequency of game meat consumption and human seropositivity (*p* = 0.7670).

Consumption of cured game meat was associated with participants’ occupational duty (*p* = 0.03); hunters were significantly more likely to eat cured game products than individuals in the “other” category (OR 6.15, 95% CI 1.45–26.10) [33]. Although individuals who consumed cured game meat showed lower seropositive rate, the difference was not statistically significant (*p* = 0.6752).

An association was observed between participants’ duties and the practice of making game meat sausages (*p* = 0.00004), with hunters being 5.5 times more likely to prepare sausages compared to park rangers (OR: 5.5, 95% CI 1.42–21.3). Furthermore, participants who frequently consumed cured meat reported making raw sausages more often (*p* < 0.0001, OR: 62, 95% CI 11.47–334). No statistically significant differences were found between study groups regarding the method used to prepare raw sausages (Fisher *p* > 0.9999). Most hunters (17/20, 80.95%) reported using a mixture of axis deer and wild boar meat, whereas park rangers exhibited more variability using either deer meat alone (2/4, 50%), a combination of deer and pork (1/4, 25%) or mixture of deer and wild boar meat (1/4, 25%).

## 4. Discussion

HEV is a major cause of acute viral hepatitis worldwide [42]. While domestic pigs and wild boar are the most important asymptomatic reservoirs for HEV-3 and HEV-4, there is a growing concern over their zoonotic risk as it has been identified in multiple species, with an ever-expanding host range [43]. Nevertheless, the epidemiological relevance and the role of potential animal hosts or reservoirs, such as deer, rats, companion animals and livestock, remain a matter of debate [17].

This study provides novel serological evidence of HEV exposure in several species, including wild boar and axis deer, within a shared ecosystem in northeastern Argentina, as well as in game meat consumers—dogs and humans. The on-site slaughtering and informal distribution of wild game meat for human consumption, together with the use of meat trimmings and offal to feed dogs, represent practices that may increase the risk of HEV exposure. Such practices facilitate close contact between humans, domestic animals, and wildlife reservoirs under non-regulated sanitary conditions and should be considered when interpreting serological findings. Notably, the observed high seropositivity rate of HEV in wild boar (38.67%) is consistent with HEV circulation in this protected area and warrants further studies to clarify their epidemiological role. In this study, no significant association between HEV seropositivity and age or sex was detected, which may be partly explained by limited statistical power related to sample size and population structure. Although no association was found here, higher HEV seroprevalence in wild ungulates has been reported, suggesting that age may influence exposure through cumulative contact over time [44,45,46]. The seropositivity rate observed in wild boar in this study is considerably higher than that reported in central Argentina, 19.6% (20/102) [30], as well as in neighboring countries, such as Uruguay (20.1%, 31/140) [38], based on the same commercial ELISA kit as this study, and also higher than findings from Brazil (13.1%, 8/61), where a different ELISA kit was used [47]. Interestingly, our results are comparable to the higher HEV seropositivity rates reported in Europe, which range from 4.9% to 34% [48], in countries such as Belgium (34%; 130/383), France (29.2%; 101/346), and Italy (28.3%; 39/138) [49,50,51]; however, differences in serological assays limit direct comparisons with these studies. Overall high seropositivity in wild boar globally emphasizes the relevance of wild boar in HEV epidemiology and the need for systematic wildlife surveillance in South America. The higher exposure levels in wild boar in EPNP likely result from their social behavior, broad habitat use, and omnivorous scavenger habits, which increase their exposure to environmental sources of HEV, such as contaminated water, feces and infected carcasses. This is particularly relevant because wild boar populations are rapidly growing and expanding their geographic range and abundance in South America [52,53]. However, it should be noted that the present study is based on serological evidence, and therefore inferences regarding viral circulation and reservoir status are indirect and should be interpreted with caution. Molecular detection of HEV RNA is required to confirm active infection and viral shedding in wildlife populations.

Environmental factors likely influence the transmission and persistence of HEV among wildlife populations [54]. In this study, we found a spatial cluster of high seropositivity of wild boar in a low-lying area prone to flooding where El Palmar stream discharges into the Uruguay river. Although the identified spatial cluster showed borderline statistical significance, it should be interpreted as hypothesis-generating rather than confirmatory, and used to guide further targeted investigation in areas where environmental conditions may favor HEV exposure. Given that HEV is mainly transmitted via fecal–oral route, often through contaminated water, aquatic environments may serve as reservoirs that facilitate indirect exposure among wildlife [55]. The spatial clustering observed in this study could reflect both the congregation of animals around water sources and the environmental dispersion of the virus, particularly in flood-prone areas where HEV persistence could facilitate indirect transmission to sympatric species (e.g., native capybaras (*Hydrochoerus hydrochaeris*)) [56].

Additionally, the El Palmar stream may become contaminated along its course through contact with animal feces. Aquatic environments have been proposed as potential reservoirs facilitating indirect HEV transmission among wildlife and may also contribute to environmental exposure. In EPNP, direct human contact with the El Palmar stream is minimal, as swimming and other recreational activities are not permitted. Importantly, the stream originates outside the park boundaries and flows through agricultural and livestock areas before entering the protected area, suggesting that environmental contamination may occur upstream and be introduced via hydrological connectivity. In addition to environmental exposure, direct animal-to-animal transmission among wild boar should also be considered, as this route has been reported in domestic pigs [57]. Because the serological assay used covers all HEV genotypes, the source of infection (whether zoonotic or anthroponotic) remains uncertain, highlighting the need for molecular studies to identify circulating viral strains and clarify transmission pathways within this ecosystem. Evidence supporting the environmental persistence of HEV comes from multiple studies reporting the detection of HEV-3 and HEV-4 in both natural surface waters (rivers and streams) and household wastewater across Europe [4] and South America [27]. In Argentina, HEV-3 RNA has also been identified in wastewater, indicating active viral circulation in urban settings from several provinces [25,58]. Nevertheless, most available data come from human-influenced environments, emphasizing the need to investigate HEV persistence and transmission dynamics in natural ecosystems. Assessing the presence of viral RNA in surface waters from protected areas such as EPNP could provide valuable insights into the environmental persistence of HEV and the potential exposure pathways for wildlife populations. Future research should not only include viral surveillance in surface waters but also determine whether wild boar excrete HEV in feces, as this would confirm active infection and provide direct evidence of their contribution to environmental contamination, as well as the potential persistence of HEV within EPNP.

The low seropositivity rate observed in axis deer (0.75%) may reflect species-specific differences in susceptibility, ecology, and habitat use. In EPNP, axis deer preferentially occupy ecotonal areas with intermediate vegetation cover, often near plantation forests and permanent watercourses such as the Uruguay river [59]. Given that their occupancy declines with increasing distance from main watercourses, the spatial ecology of axis deer likely contributes to the lower seroprevalence detected. Nevertheless, the detection of only one seropositive individual should be interpreted with caution but may indicate previously undetected HEV exposure in this species in Argentina. Recently, in Uruguay, HEV seropositivity was reported in 11.1% (6/54) of free-ranging axis deer, along with the detection of HEV-3 in fecal samples, suggesting that this invasive species may play a role in HEV transmission in the area [27]. The seroprevalence reported in that study is consistent with previous reports in other Cervidae species across Europe and North America, with prevalences ranging from 1.4 to 19% [15,60,61,62]. Given their abundance and co-existence with wild boar in EPNP, the inclusion of deer in surveillance programs is recommended due to their potential role as spillover hosts within this ecosystem. Moreover, the role of axis deer and other cervids in HEV transmission, particularly in areas where they are sympatric with wild boar, deserves further study.

Carnivores, including dogs, are not considered reservoirs of HEV, as evidence of sustained viral replication and viral shedding in these species is scarce. Consequently, the detection of total anti-HEV antibodies in dogs is generally interpreted as evidence of prior exposure to the virus rather than ongoing viral replication [19,63,64]. In the present study, the detection of anti-HEV antibodies in one dog (5.5%) provides preliminary evidence of HEV exposure in canines in Argentina but should be interpreted with caution given the small sample size and the limitations of serological assays. Nevertheless, our finding is consistent with previously reported HEV prevalence in hunting dogs in Italy (5.5%; 4/80) [63]. Anti-HEV antibodies have been reported in dogs on different continents, with seroprevalences ranging between 0.9% and 56.6% [19]. Although infection in dogs is generally asymptomatic and viral RNA has not yet been detected [63,64,65], their frequent contact with wildlife and carcasses during hunting-related activities makes them valuable sentinels of environmental HEV contamination [19]. In our study, owners reported feeding dogs with offal and raw meat from hunted animals, suggesting a potential transmission route linking the wildlife source with companion animals, and possibly humans, through carcass dressing and food preparation practices. These findings underscore the importance of implementing biosecurity measures in both food handling and animal care within the hunting community.

In this context, human serological findings provide complementary information to the wildlife results, contributing to a broader understanding of HEV exposure in a shared ecosystem. In Argentina, reported human HEV seroprevalence shows geographic and temporal variability. Very low levels have been described in non-endemic areas (1.80%) [66], whereas more recent studies from northwestern regions reported higher seroprevalence ranging from 5.6% to 9% [67,68]. In central Argentina, an HEV seroprevalence of 3.47% has been reported among blood donors, whereas higher and more heterogeneous rates, ranging from 5.1% to 20%, have been observed across five regions of the country [69]. It should be noted that most of these previous studies in Argentina relied on ELISA assays detecting anti-HEV IgG antibodies only, whereas the present study used an ELISA that detects total anti-HEV antibodies (IgG/IgM/IgA), which may partially account for differences in reported rates. In this context, the human seropositivity rate observed in EPNP (10.17%) aligns with the upper range of previously reported values. Although none of the participants reported a history of hepatitis, asymptomatic or misdiagnosed infections cannot be ruled out. Despite the lack of a statistically significant association between seropositivity and game meat consumption—possibly due to the limited sample size—high-risk practices such as sausage preparation and the consumption of cured or undercooked meat were frequently reported. Foodborne HEV transmission by consumption of raw or undercooked pork, wild boar, or deer meat has been well-documented [70,71], as has occupational exposure among veterinarians, slaughterhouse workers, hunters and farmers [72,73,74]. The HEV can persist in food products for extended periods, particularly under refrigeration or freezing [75]. This raises concerns about foodborne HEV transmission, not only via eating game meat but also cross-contamination of surfaces during food preparation. To ensure effective inactivation of the virus, meat should be cooked to an internal temperature of at least 71 °C [76,77]. These observations are consistent with wildlife-associated exposure pathways and support the relevance of integrating veterinary, environmental, and public health perspectives in HEV surveillance.

Several findings in this study are based on limited sample sizes across species, which may have reduced the statistical power to detect significant associations; therefore, the absence of statistically significant relationships should be viewed with caution and not as evidence of the absence of an association. In this study, HEV antibodies were detected using a commercial ELISA kit based on a recombinant ORF-2 encoded protein, which is highly conserved in all HEV genotypes [78]. This ELISA assay has been previously validated and widely used in studies involving multiple host species, demonstrating robust performance and serving as a reference for other immunoassays [27,28,38]. Nevertheless, the true sensitivity and specificity of multi-species ELISA assays may vary among different species and remain uncertain. In addition, although this method is practical for large-scale serosurveys, it cannot differentiate between recent and past infections in either humans or animals. Moreover, the serological nature of this research, without molecular identification of the genotypes involved, limits interpretation of transmission dynamics between species as well as environmental sources. Therefore, future studies should incorporate molecular and genomic surveillance to identify circulating HEV strains, assess viral shedding in different hosts, and better characterize potential transmission pathways. Environmental monitoring of watercourses and wetlands within EPNP is recommended to improve understanding of the local epidemiology of HEV.

## 5. Conclusions

In conclusion, this study suggests HEV exposure in multiple species within a protected ecosystem and that current wildlife management practices may be increasing risk of human exposure. The high seropositivity in wild boar, coupled with evidence of exposure in other species, highlights the need for targeted surveillance and improved health education for hunters and consumers to reduce zoonotic risks and protect public health in the region. Together with our previous studies on other zoonotic agents in EPNP [33,37], these results emphasize the importance of sustained and integrated health surveillance. Incorporating HEV monitoring into invasive alien species management programs would provide a cost-effective approach to prevent zoonotic risk and contribute to One Health strategies in Argentina.

## Figures and Tables

**Table 1:** Summary of serological status for HEV in wildlife: wild boar (*Sus scrofa*) related to sex and age categories and location of watchtower where animals were killed in El Palmar National Park, Entre Ríos, Argentina, 2017–2019.

Variable	Category	Positive/Tested	% (95% CI)	*p* Value
Sex	Female	15/30	50.0 (31.3–68.7)	
	Male	14/45	31.1 (18.2–46.6)	0.0980
Age class *	Juvenile	13/36	36.1 (20.8–53.8)	
	Young adult	6/15	40.0 (16.3–67.7)	
	Mature adult	3/8	37.5 (8.5–75.5)	
	Older adult	7/16	43.8 (19.8–70.1)	0.9623
Sampling area	North	17/39	43.6 (27.8–60.4)	
	South	12/36	33.3 (18.6–51.0)	0.2505

* Individuals were classified into four age categories according to body length and sex, following the criteria described [31].

**Table 2:** Comparison of median distances to the Uruguay river and other watercourses, and mean altitude between seronegative and seropositive wild boar culled in El Palmar National Park, Entre Ríos, Argentina, 2017–2019.

Variable	Seropositives Median (Q1–Q3)	Seronegatives Median (Q1–Q3)	Wilcoxon Test *p* Value
Distance to (m) Uruguay river	3326.98 (2276.13–4638.85)	6101.62 (1910.74–8664.92)	0.0611
Distance to (m) El Palmar stream	681.08 (431.74–2014.35)	1191.01 (681.08–1834.61)	0.1800
Distance to (m) Los Loros stream	3320.13 (1417.19–3983.23)	4115.31 (1407.97–4601.35)	0.2402
Distance to (m) Espina stream	2647.52 (1726.44–4946.97)	3180.44 (1270.18–4946.97)	0.7312
Altitude (m.a.s.l.)	22 (15–24)	24 (22–26)	0.0176

**Table 3:** Summary of demographic characteristics, game meat consumption and anti-HEV serological results among participants involved in invasive species control at El Palmar National Park, Argentina (2019).

Category	Variable	Hunters (*n* = 31)	Park Rangers (*n* = 14)	Other (*n* = 14)	Total (*n* = 59)
Demographics	Male sex	28 (90.3%)	10 (71.4%)	10 (71.4%)	48 (81.4%)
	Female sex	3 (9.7%)	4 (28.6%)	4 (28.6%)	11 (18.6%)
	Age (mean ± SD, range)	42.3 ± 11 (24–67)	43.1 ± 7.5 (35–58)	35.9 ± 11.5 (27–50)	41 ± 10.6 (24–67)
Game meat consumption frequency *	Very low	3 (9.7%)	2 (14.3%)	6 (42.9%)	11 (18.6%)
	Low	15 (48.4%)	9 (64.3%)	5 (35.7%)	29 (49.2%)
	Medium	6 (19.3%)	1 (7.1%)	3 (21.4%)	10 (16.9%)
	High	7 (22.6%)	2 (14.3%)	0 (0%)	9 (15.3%)
Preparation of raw game meat sausages	Deer and wild boar	16 (51.6%)	1 (7.1%)	0 (0%)	17 (28.8%)
	Deer and pork	3 (9.7%)	1 (7.1%)	0 (0%)	4 (6.8%)
	Deer	1 (3.2%)	2 (14.3%)	0 (0%)	3 (5.1%)
	No	11 (35.5%)	10 (71.4%)	14 (100%)	35 (59.3%)
Feed game meat/offal to dogs	Raw meat	19 (61.3%)	8 (57.1%)	6 (42.9%)	33 (55.9%)
	Cooked meat	4 (12.9%)	3 (21.4%)	4 (28.6%)	11 (18.7%)
	No	8 (25.8%)	3 (21.4%)	4 (28.6%)	15 (25.4%)
HEV seropositivity **	-	3 (5.08%)	1 (1.69%)	2 (3.38%)	6 (10.17%)

* Consumption categories defined as: very low (<1/month), low (1–3/month), medium (1–2/week), high (≥3/week). ** Commercial kit ELISA HEV Ab version Ultra; DIA.PRO multi species (Milan, Italy).

## Data Availability

The original contributions presented in this study are included in the article. Further inquiries can be directed to the corresponding author.

## Abbreviations

The following abbreviations are used in this manuscript:

HEV	Hepatitis E Virus
WHO	World Health Organization
RNA	Ribonucleic acid
EPNP	El Palmar National Park
ELISA	Enzyme-Linked Immunosorbent Assay
